# Transcription of Human Resistin Gene Involves an Interaction of Sp1 with Peroxisome Proliferator-Activating Receptor Gamma (PPARγ)

**DOI:** 10.1371/journal.pone.0009912

**Published:** 2010-03-29

**Authors:** Anil K. Singh, Aruna Battu, Krishnaveni Mohareer, Seyed E. Hasnain, Nasreen Z. Ehtesham

**Affiliations:** 1 University of Hyderabad, Hyderabad, India; 2 National Institute of Nutrition, Indian Council for Medical Research, Hyderabad, India; 3 Institute of Life Sciences, University of Hyderabad, Hyderabad, India; 4 Jawaharlal Nehru Centre for Advanced Scientific Research, Bangalore, India; Charité-Universitätsmedizin Berlin, Germany

## Abstract

**Background:**

Resistin is a cysteine rich protein, mainly expressed and secreted by circulating human mononuclear cells. While several factors responsible for transcription of mouse resistin gene have been identified, not much is known about the factors responsible for the differential expression of human resistin.

**Methodology/Principal Finding:**

We show that the minimal promoter of human resistin lies within ∼80 bp sequence upstream of the transcriptional start site (−240) whereas binding sites for cRel, CCAAT enhancer binding protein α (C/EBP-α), activating transcription factor 2 (ATF-2) and activator protein 1 (AP-1) transcription factors, important for induced expression, are present within sequences up to −619. Specificity Protein 1(Sp1) binding site (−276 to −295) is also present and an interaction of Sp1 with peroxisome proliferator activating receptor gamma (PPARγ) is necessary for constitutive expression in U937 cells. Indeed co-immunoprecipitation assay demonstrated a direct physical interaction of Sp1 with PPARγ in whole cell extracts of U937 cells. Phorbol myristate acetate (PMA) upregulated the expression of resistin mRNA in U937 cells by increasing the recruitment of Sp1, ATF-2 and PPARγ on the resistin gene promoter. Furthermore, PMA stimulation of U937 cells resulted in the disruption of Sp1 and PPARγ interaction. Chromatin immunoprecipitation (ChIP) assay confirmed the recruitment of transcription factors phospho ATF-2, Sp1, Sp3, PPARγ, chromatin modifier histone deacetylase 1 (HDAC1) and the acetylated form of histone H3 but not cRel, C/EBP-α and phospho c-Jun during resistin gene transcription.

**Conclusion:**

Our findings suggest a complex interplay of Sp1 and PPARγ along with other transcription factors that drives the expression of resistin in human monocytic U937 cells.

## Introduction

Resistin/FIZZ3 (Found in Inflammatory Zone) is a novel cysteine rich hormone expressed in the white adipose tissue in mice and in mononuclear cells in humans [1]. In genetic and diet induced mouse models of obesity resistin levels were elevated and therefore was concluded to be a link between obesity and insulin resistance [2]. Resistin is expressed at very low levels in human adipocytes and does not seem to correlate with insulin resistance [3]–[9]. Interestingly, human resistin protein is detected at a high level in mononuclear cells [4], [10] and has been shown to be upregulated in inflammation. Also, a direct correlation between inflammatory markers and resistin is evident in humans [11]–[15]. Striking similarities exist between adipocytes and macrophages and these include the secretion of many pro-inflammatory cytokines and chemokines such as Tumor Necrosis Factor-α (TNF-α), interleukins, Monocyte chemotactic protein-1, Matrix metalloproteases, by the adipocytes [16], [17]. Also, adipocytes are sensitive to stimulation with LPS (Lipopolysaccharide) and TNF-α, a characteristic feature of macrophages. Conversely, many of the proteins secreted by adipocytes such as leptin, adiponectin and resistin are also involved in inflammatory responses [18], [19]. Transcription factor PPARγ, which is specific to adipocytes, has been shown to be important in macrophages as well [20]. Thus, it is evident that a considerable overlap exists, possibly due to the common mesodermal origin, between the adipose tissue and macrophages.

Substantial evidences exist supporting the functional differences between human and mouse resistin proteins. It is also likely that, different regulatory mechanisms for the human and mouse resistin gene transcription may be expected. There are a few reports to demonstrate the role of C/EBP-α in the regulation of both human and mouse resistin genes [21]–[23]. A 224 bp segment of the mouse resistin gene promoter carries the C/EBP-α binding site, which is necessary and sufficient for transcription from the resistin gene promoter. Furthermore, C/EBP-α binding was associated with the recruitment of co-activators p300 and CREB-binding protein [21]. Human resistin gene (*hres*) promoter was also activated by C/EBP-α expression in mouse 3T3L1 cells [23]. It was further shown that thiazolidinediones (TZD) and non-TZD PPARγ ligands down-regulate resistin gene expression. Further, Retinoid X receptor (RXR) agonists, which activate the PPAR/RXR heterodimer, downregulate resistin gene expression in 3T3L1 adipocytes [21], [24]–[25]. We earlier showed that the PPARγ response element (PPRE) present in the intron X of mouse resistin gene governs the TZD responsiveness of resistin gene expression [26]. Downregulation of resistin in 3T3L1 cells has also been reported to be signaled *via* the PI3 and MAP kinase pathways [24]. Furthermore, one of the adipogenic transcription factor, adipocyte determination and differentiation dependent factor 1/sterol regulatory element-binding protein 1c (ADD1/SREBP1c) was shown to bind to the *hres* gene promoter and ectopic expression of ADD1/SREBP1c significantly increased the expression of resistin mRNA in mouse adipocytes [23]. However, another report demonstrated that SREBP1c and cyclic AMP response-element- binding protein (CREB) had no effect on resistin gene expression in 3T3L1 preadipocytes [25]. These discrepancies in the function of various transcription factors in resistin gene regulation might be due to the variation in the system used by the different investigators. Hence it becomes pertinent to understand the possible role of different transcription factors mediating the regulation of the expression of resistin where it is highly expressed, namely the monocytes/macrophages.

Despite the well documented involvement of PPARγ in regulating *hres* expression in human macrophages, paradoxically PPARγ binding site has not been found within the *hres* regulatory sequences. We highlight the role of transcription factors responsible for *hres* expression in the human monocytic cell line, U937. Our findings suggest that Sp1 is a crucial transcription factor that drives the expression of human resistin in U937 cells. We also find that PMA and LPS increase the level of *hres* mRNA while pyrrolidine dithiocarbamate (PDTC), which is an inhibitor of Sp1 binding, abrogates the basal, and also PMA/LPS induced resistin mRNA levels. Furthermore, PPARγ plays an important role in the regulation of *hres* gene expression by interacting directly with Sp1 transcription factor.

## Materials and Methods

### Cloning of the promoter sequences

The amplicon corresponding to +32 to −673 region of *hres* gene was cloned into pGL3Basic vector using *Kpn*I and *Hin*dIII restriction sites (pGL3*hres*0.7K). pGL3*hres*0.65K corresponding to the region −630 to +32 region was generated by digesting pGL3*hres*0.7K with *Kpn*I and *Sma*I followed by end filling and self-ligation. The amplicon corresponding to −238 to +32 was cloned into *Kpn*I and *Hin*dIII sites of pGL3Basic (pGL3*hres*0.25K). The PCR amplified fragment from −673 to −284 was cloned in *Kpn*I and *Nco*I enzyme sites in pGL3Basic (pGL3*hres*0.4K). The −1262 to −284 region and −2140 to −284 promoter regions were amplified and primarily cloned in pCR2.1 vector (Invitrogen) followed by sub cloning in pGL3Basic into *Hin*dIII *Xho*I and *Xho*I *Kpn*I sites to generate pGL3*hres*1K and clones, respectively. The pGL3*hres*0.7K construct was digested with *Kpn*I and *Apa*I and the plasmid re-ligated to generate pGL3*hres*0.34K construct. pGL3*hres*0.39K construct was generated by PCR-amplification of −367 to +32 region of resistin gene using pGL3*hres*0.7K plasmid as the template and the amplicon inserted into *Kpn*I and *Hin*dIII sites of pGL3Basic vector.

### Prediction of transcription factor binding sites regulating human resistin gene expression

Transcription factor binding sites in human resistin gene promoter were predicted using Matinspector algorithm (http://www.genomatix.de and http://www.cbrc.jp/research/db/TFSEARCH.html).

### Site-directed mutagenesis of promoter constructs

Promoter constructs with site-directed mutations of the various transcription factor binding sites in *hres* gene promoter were generated using pGL3*hres*0.7K plasmid as the basic plasmid. A detailed description of the methodology is given in the Text S1.

### Transient transfection of promoter clones

Transfection of U937 cells was performed using Electroporator (Bio-Rad) using exponential decay method. 25×10^6^ cells were electroporated with 40 µg reporter plasmid and 10 µg pSVβGAL (Promega) plasmid at 300 V and 950 µF. Cells were harvested 24 or 48 hours post transfection and assayed for luciferase and β-Gal reporter enzymes using the luciferase and β-Gal assay systems from Promega as per the manufacturer's instructions. Experiments were repeated at least 3 times.

### Stimulation of U937 cells

U937 cells (6×10^6^) were treated with 10 nM, 20 nM and 50 nM PMA. The cells were also stimulated with either 100 nM PMA, 1 mM PDTC, 5 µg/ml LPS alone or PDTC in combination with PMA and LPS for 3 hours. RNA was isolated and quantitative RT-PCR was performed to monitor resistin mRNA level.

### Electrophoretic mobility shift assay (EMSA)

#### Preparation of Nuclear Extracts

Nuclear extracts were prepared according to the method described by Dignam *et al* [27]. Briefly, cells were washed three times with ice- cold PBS containing 1 mM PMSF. Lysis buffer (30 mM Tris pH 7.5, 1 mM Mg-acetate, 1% NP- 40, 1 mM PMSF) was added at five times the packed cell volume (PCV) and vortexed. Nuclei were harvested by centrifugation at 1600 rpm at 4°C. The supernatant was discarded and four PCVs of nuclear extraction buffer (10 mM HEPES-KOH pH 7.5, 420 mM NaCl, 1.5 mM MgCl_2_, 10% glycerol, 1 mM PMSF, 1 mM DTT, 3 µg/ml Leupeptin, 1 µg/ml Aprotinin) was added. The tubes were incubated on ice for 60 minutes with gentle shaking. The debris was removed by centrifugation at 13000 rpm for 10 minutes at 4°C and the supernatant containing the nuclear extract was stored in small aliquots at −70°C. Protein concentration was estimated as described [28].

#### Preparation of Radio labeled Probe

100 ng of the respective oligonucleotides were end labeled using 5 units of polynucleotide kinase (NEB) in 1X buffer with P^32^ γ-ATP for one hour at 37°C. The reaction was stopped by the addition of EDTA and the labeled probe was purified using a G-50 column.

#### Electrophoretic mobility shift assay

EMSAs were performed using nuclear extract from U937 cells. The reaction mix contained 7.5 µg of nuclear protein, 1 ng of labeled oligonucleotide, 1 µg poly dIdC and 1X EMSA binding buffer. The compositions of the binding buffers for the transcription factors AP-1: 50 mM Tris-Cl, pH 8.0, 12.5 mM MgCl_2_, 1 mM EDTA, 1 mM DTT, 10% glycerol; c-Rel: 10 mM Tris-Cl, pH 7.5, 100 mM NaCl, 1 mM EDTA, 5 mM DTT, 100 µg/ml BSA, 4% glycerol, and Sp1: 10 mM Tris-Cl, pH 7.5, 75 mM KCl, 0.1 mM EDTA, 2.5 mM MgCl_2_, 0.25 mM DTT, 10% glycerol. The authenticity of binding was confirmed using cold competitors, both specific (self or consensus) as well as non-specific oligonucleotides.

For super-shift of protein-DNA complexes, 2 µg of respective antibodies were used in binding reaction. The reaction mixture was incubated on ice for 20 minutes with the cold competitor, followed by incubation with labeled oligonucleotide for another 30 minutes. After electrophoresis on 7% TGE-polyacrylamide gel, the gel was dried and autoradiographed in a phosphorimager.

### Chromatin Immuno- Precipitation assay (ChIP)

U937 cells were seeded at a concentration of 1×10^6^/ml in a 100 mm tissue culture dish in RPMI 1640 supplemented with 10% FBS. Approximately 25×10^6^ cells were stimulated with 5 ng/ml PMA for 5 hours. Cross-linking was performed by adding formaldehyde to a final concentration of 1% at room temperature for 10 minutes and reaction stopped by the addition of 125 mM glycine. Cells were washed with ice-cold phosphate buffered saline containing 0.1 mM PMSF. Cell pellets, collected by centrifugation at 2000 rpm at 4°C, were resuspended in 1 ml of ChIP sonication buffer (1% Triton X-100, 0.1% Deoxycholate, 50 mM Tris-HCl, pH 8.1, 150 mM NaCl, 5 mM EDTA, 0.1 mM PMSF, 2 µg/ml aprotinin and leupeptin) and placed on ice for 10 minutes. DNA was sheared by sonication and the cell debris was pelleted by centrifugation at 14,000xg for 15 minutes. The supernatant was collected in fresh tubes and pre cleared with Protein G sepharose equilibrated with salmon sperm DNA for 30 minutes at 4°C. The pre-cleared whole cell extract was incubated with or without the antibodies, as described in the legend, at 4°C overnight. 10% of the volume was frozen and kept aside as input before the addition of the antibodies.

The immune complex was precipitated by incubation of the samples with Protein G sepharose equilibrated with salmon sperm DNA at 4°C for 2 hours. The samples were centrifuged at 2500 rpm at 4°C for 1 minute and the supernatant was discarded. The Protein G sepharose beads were washed with cold ChIP sonication buffer containing no protease inhibitors. This was followed by sequential washes with low salt wash buffer (1% Triton X-100, 0.1% sodium deoxycholate, 50 mM Tris pH 8.0, 150 mM NaCl and 5 mM EDTA), high salt wash buffer (1% Triton X-100, 0.1% sodium deoxycholate, 50 mM Tris pH 8.1, 500 mM NaCl and 5 mM EDTA) and LiCl wash buffer (0.25 M LiCl, 0.5% NP-40, 0.5% sodium deoxycholate, 1 mM EDTA and 10 mM Tris pH 8.1). The immune complex was eluted with 1% SDS, 0.1 M NaHCO3. The cross linking was reversed by incubation at 65°C for 4 hours followed by ethanol precipitation of the proteins and DNA. Proteinase K digestion was performed at 55°C for 1 hour. The immunoprecipitated DNA was extracted by phenol:chloroform. PCR was performed using forward (5′ TGTCATTCTCACCCAGACACA 3′) and reverse (5′ TGGGCTCAGCTAACCAAATC 3′) primers to generate a specific 535 bp fragment corresponding to the *hres* gene promoter.

### RT-PCR for human resistin

Human resistin was amplified by RT- PCR with total RNA isolated from U937 cells using Trizol (Invitrogen). Upstream (5′ CG AGA TCT ATG AAA GCT CTC TGT CTC CTC CTC G 3′) and downstream (5′ GGA ATT CCC TCA GGG CTG CAC ACG ACA 3′) primers were used for RT-PCR which was performed in a GeneAmpR PCR system 9700 (Applied Biosystems) using Access RT-PCR system (Promega Corporation) with the following cycles: 48°C for 45 minutes; 94°C for 2 minutes; followed by 30 cycles of 94°C, 58°C, 68°C for 30 seconds each and finally 72°C for 7 minutes. RT-PCR of GAPDH was used as an internal control. The amplified products were resolved on a 1.5% agarose gel and the intensity of bands analyzed using Quantity One software (Bio-Rad).

### Immuno-precipitation

U937 cells were washed in PBS and centrifuged at 800 to 1000 rpm for 5 minutes to harvest the cells. Ice-cold modified RIPA buffer (50 mM Tris-HCl, pH 7.4, 1% NP-40, 0.25% Na- deoxycholate, 150 mM NaCl, 1 mM EDTA, 1 mM PMSF, 1 µg/ml each of aprotinin, leupeptin, pepstatin, 1 mM Na_3_VO_4_, 1 mM NaF) was added to cells and gently rocked on a rocker at 4°C for 15 minutes for cell lysis. The lysate was centrifuged at 14,000xg for 15 minutes at 4°C. The cell lysate was pre-cleared by adding 100 µl of 50% Protein G sepharose bead slurry per 1 ml of cell lysate. The supernatant was incubated with specific antibody overnight at 4°C with gentle rocking. The immune complex was captured by the addition of 100 µl Protein G sepharose. The sepharose beads were washed 3 times with 800 µl ice-cold RIPA buffer and resuspended in 60 µl 2× sample buffer, followed by elution through boiling. The immune complexes were fractionated on 12% SDS-PAGE followed by western blotting with appropriate antibodies.

### ‘Immuno-pullout’ of specific transcription factors from nuclear extracts

Nuclear extract from U937 cells was dialyzed against 1× Sp1 binding buffer at 4°C for one hour. Protein concentration of the dialyzed nuclear extracts was determined by the Bradford method. 30 µg of the dialyzed nuclear extracts were incubated with 2 µg each of anti-Sp1, anti- Sp3 and anti-PPARγ antibodies along with Protein G sepharose beads for 4 hours at 4°C with gentle shaking. The supernatants were collected by centrifugation at 5000 rpm for 2 minutes at 4°C. 7.5 µg of the supernatant was used to perform EMSA as described earlier.

## Results

### Delineation of the human resistin gene promoter sequences

**Figure 1:**
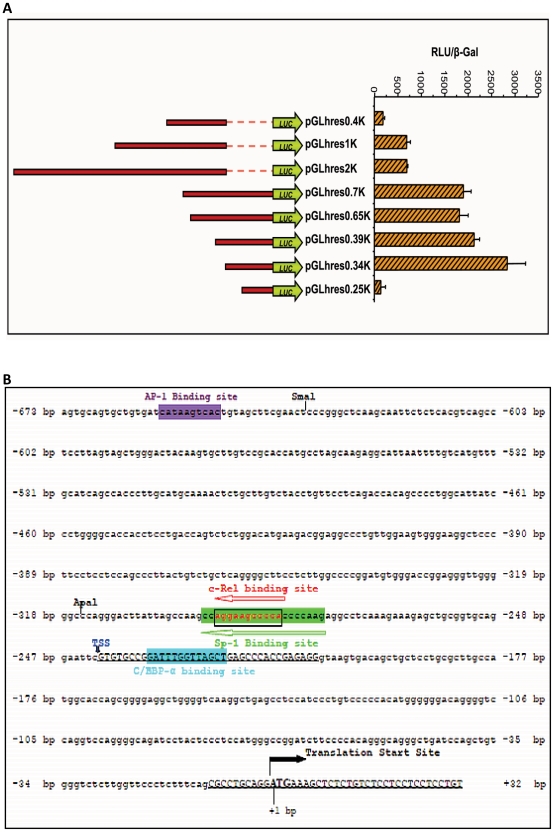
Delineation of the optimal promoter of human resistin. A. The regulatory sequences of *hres* gene were cloned upstream of luciferase (luc) reporter gene in pGL3Basic vector. These constructs were transfected into U937 cells along with pSVβ-Gal plasmid and expression of luciferase gene was assayed 48 hours post transfection. B. Putative transcription factor binding sites in human resistin gene. The sequence of the *hres* gene promoter is shown and numbered relative to the first ATG. The exons 1 and 2 sequences are underlined. The transcription start site (TSS) is indicated by a blue arrowhead and the translation start site by an arrow. AP-1, c-Rel, Sp1 and C/EBP-α binding sites are indicated.

As a first step in understanding the regulation of *hres* gene expression the DNA sequence elements required to drive the expression of human resistin in U937 cells were delineated. Different plasmid constructs carrying defined regions upstream of the human resistin translation start site were generated in pGL3 Basic reporter plasmid (Promega) (Figure 1A). The various deletion constructs of the putative promoter were transfected into U937 cells along with pSVβGal vector in a ratio of 8∶2. Cells were harvested at 24 and 48 hours post transfection and luciferase and β-galactosidase assays were performed. Relative luciferase units (RLU) normalized to β-Gal activity for 48 hours post transfection are presented in Figure 1A. pGL3*hres*0.34K construct exhibited maximal expression, indicating that the optimal promoter lies within 317 nucleotide upstream of the initiating ATG. Negligible luciferase gene expression was observed in cells transfected with pGL3*hres*0.25K (Figure 1A) suggesting that the optimal promoter that drives the expression of human resistin might be present within the 80 nucleotide stretch that is absent in pGL3*hres*0.25K construct. pGL3*hres*0.7K, pGL3*hres*0.65K pGL3*hres*0.39K showed significant luciferase reporter gene expression albeit lesser than pGL3*hres*0.34K (Figure 1A). Sequences further away from −340 contain possible negative regulatory elements so much so that the region extending up to −2300 nucleotide upstream showed negligible expression of luciferase reporter gene (data not shown). Constructs pGL3*hres*2K, pGL3*hres*1K and pGL3*hres*0.4K, which are devoid of 284 bp upstream of ATG, were not able to drive the expression of luciferase reporter gene. These results point towards the importance of the *cis*- acting elements present in the region between translation start site and -673 in driving resistin gene expression *in vivo*. More particularly the sequence between nucleotide −316 to +32 appears to be critical for resistin gene transcription.

### Prediction of transcription factor binding sites in the 5′ flanking regions of human resistin gene

In order to identify the trans-acting factors involved in regulating resistin gene transcription, *in silico* analysis of the upstream 673 bp sequence of *hres* gene using the Matinspector software (http://www.genomatix.de and http://www.cbrc.jp/research/db/TFSEARCH.html) was carried out. The presence of a number of transcription factor binding sites (Figure 1B) which included c-Rel (−281 to −294), C/EBP-α (−221 to −233), CREBP-1/ATF -2 (−599 to −619), AP-1 (−647 to −657) and Sp1 (−276 to −295) that might be potential regulators of constitutive and induced expression of resistin in human monocytic cells could be seen. Interestingly, the absence of TATA box element within the human resistin gene promoter points to the possibility of resistin gene transcription being regulated by an “initiator promoter” [29]–[32].

### Sp1 binds specifically to its cognate motif present within the human resistin gene promoter

**Figure 2:**
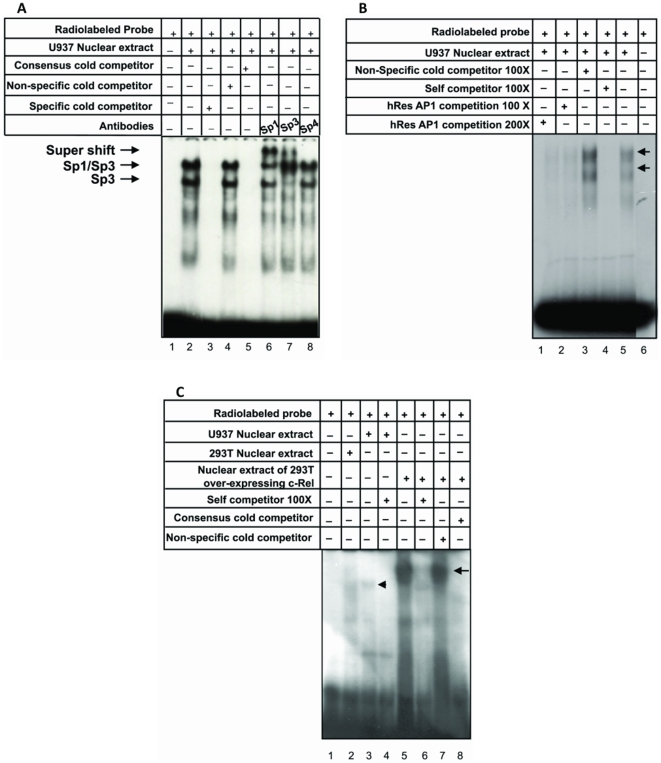
Sp1, AP-1 and c-Rel bind to their cognate motifs present within the *hres* regulatory sequences. A. Sp1 binds specifically to the *hres* cognate motif. EMSA with Sp1 binding motif using U937 cells and supershift with Sp1, Sp3 and Sp4 antibodies. EMSA using nuclear extract prepared from U937 cells shows AP-1 binding to its *hres* cognate motif (B) and weak binding of c-Rel to its *hres* cognate motif (C). Arrows indicate the position of binding of the corresponding trans-acting factors to their cognate motif and the super-shifts using antibodies. These experiments were repeated two times.

TATA-less promoters are regulated by the involvement of Sp-like factors which act as tethering agents to recruit specific transcription factors [29], [31]. We, therefore, investigated the actual interaction, if any between resistin gene promoter and Sp1 factor, more so given the presence of Sp1 binding site predicted *in-silico*. The interaction of the Sp1 binding site in human resistin gene with Sp1 transcription factor present in U937 cells was assessed by performing EMSA using radio labeled oligonucleotide corresponding to *hres* Sp1 binding motif (−276 to −295). The *hres* specific Sp1 binding sequence showed strong binding to factors present in U937 nuclear extract generating two prominent protein-DNA complexes (Figure 2A, lane 2). The binding was abolished with homologous competition using unlabeled *hres* Sp1 oligonucleotide (Figure 2A, lane 3) and also with consensus Sp1 oligonucleotide (Figure 2A, lane 5) demonstrating the specificity and authenticity of binding. There was no effect on the complex formation when binding was carried out in the presence of unlabeled polyhedrin gene promoter (Figure 2A, lane 4) [32] used as a non-specified control. To categorically demonstrate that the factor binding to Sp1 motif, present within the resistin gene, is indeed Sp1, super shift assays were carried out. It could be observed that anti-Sp1 antibodies could super-shift the protein-DNA complex (Figure 2A, lane 6) confirming the presence of Sp1 in the complex. Additionally, anti-Sp3 antibodies could also super-shift the protein-DNA complex (Figure 2A, lane 7) thereby demonstrating the presence of Sp3 factors in the protein-DNA complex. On the other hand, anti-Sp4 antibodies did not have any effect on the formation of the protein-DNA complex (Figure 2A, lane 8). These results demonstrate that the putative *hres* Sp1 binding site specifically recruits Sp1 and Sp3 transcription factors present within U937 nuclear extracts, either as a heterodimer of Sp1 and Sp3 or as a homodimer of Sp3. Furthermore the complex formation does not involve Sp4 factors.

### Endogenous AP-1 binds to its cognate motif in the human resistin gene promoter

The AP-1 transcription factor is known to be involved in regulation of genes under various conditions of stress. That resistin is pro- inflammatory molecule was earlier reported [15] thereby suggesting that human resistin could be transcriptionally regulated by factors like AP-1, the cognate motifs for which were indeed present (Fig. 1B) within the resistin gene upstream regulatory sequences (−657 to −648). Experiments were, therefore, designed to document the interaction between *hres* AP-1 motif and AP-1 factors present in U937 nuclear extracts using EMSA. Consensus AP-1 oligonucleotide was radio labeled and incubated with the nuclear extract prepared from U937 cells. As expected, the consensus AP-1 oligonucleotide could bind to AP-1 proteins present in the U937 nuclear extracts (Figure 2B, lane 5). There was no effect on the complex formation when binding was carried out in the presence of unlabeled competitor oligonucleotides carrying *hres* gene promoter sequence spanning nucleotides −221 to −233 present upstream of ATG and devoid of AP-1 recognition motif (Figure 2B, lane 3). Homologous competition using consensus AP-1 resulted in the disappearance of the AP-1 complex (Figure 2B, lane 4) pointing to the specificity of the DNA protein interaction thereby clearly indicating that the complex is not an artifact generated by non-specific protein binding. Authenticity of these complexes were established by competition analyses using 100 and 200× (Figure 2B, lanes 2 and 1, respectively) molar excess of oligonucleotides corresponding to the putative AP-1 binding site spanning −647 to −657 of the *hres* gene promoter. These results clearly demonstrate that AP-1 transcription factor recognizes the cognate AP-1 binding sequence present within the *hres* gene promoter.

### Endogenous c-Rel binds to the cognate c-Rel binding motifs in the human resistin gene promoter

The presence of c-Rel binding motif, overlapping with the Sp1 motif, within the upstream (−281 to −294) *hres* gene regulatory sequences tempted us to investigate direct binding of cRel factor to the *hres* c-Rel cognate motif. c-Rel which is a member of the Rel/NFκB family of transcription factors, forms homo- or heterodimeric complexes with other members of the family to regulate normal hemopoietic and immune cell function. Radio labeled oligonucleotide corresponding to the *hres* c-Rel binding motif (−281 to −294) was incubated with U937 nuclear extract as well as with nuclear extract from 293T cells over-expressing c-Rel. EMSA was performed to check the interaction of c-Rel with the cognate binding motifs present within the *hres* upstream DNA sequence elements. It could be observed that c-Rel sequence motif, present within the *hres* promoter, forms a complex with U937 nuclear extract and also nuclear extract from 293T cells over-expressing c-Rel (Figure 2C, lanes 3 and 5, respectively) but not when nuclear extract was prepared from normal 293T cells (lane 2). However, when U937 nuclear extract (lane 3) was used, the intensity of the complex is low as compared to the complex (lane 5) formed with the over-expressed c-Rel present in 293T cells. This is perhaps a reflection of the abundance of the c-Rel protein in the over-expressing 293T cells. It could also be seen that the size of the complex formed with U937 nuclear extract was lower as compared to that formed with nuclear extract from 293T cells over- expressing c-Rel probably pointing to the formation of P65 c-Rel homodimers in the case of 293T cells over-expressing c-Rel as compared to the formation of P50/P65-c-Rel heterodimers in U937 cells. This protein-DNA complex is abolished (Figure 2C, lane 8) upon competition with unlabelled consensus c-Rel oligonucleotide and also sequences corresponding to homologues *hres* c-Rel motif (lanes 4, 6) but is unaffected in the presence of non-specific cold competition (lane 7). These results indicate that the c-Rel protein binds to putative binding site, present within −281 to −294 upstream of *hres* promoter, pointing to the likely role of c-Rel in resistin gene regulation *in vivo*.

### Sp1 transcription factor regulates the constitutive expression of human resistin in U937 cells

**Figure 3:**
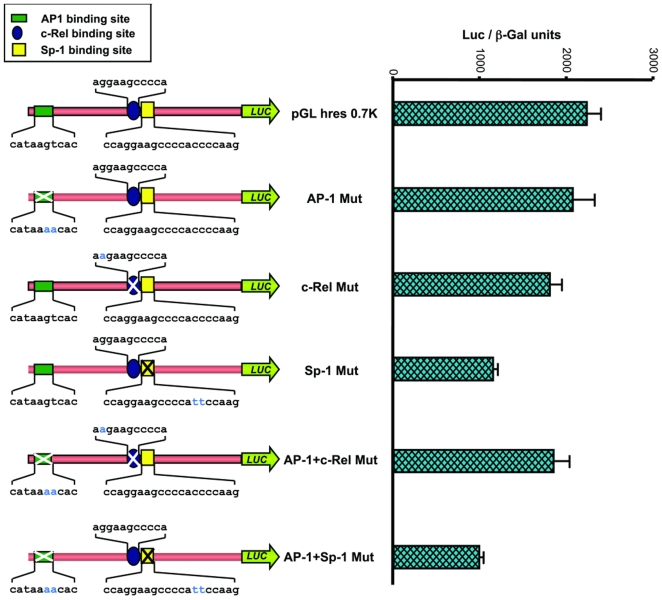
Sp1 binding is important for human resistin gene promoter activity. A schematic representation of the human resistin gene promoter constructs with mutations in the various transcription factor binding motifs is shown. The location of transcription factor binding sites is indicated by different symbols given in the inset. Mutated bases are indicated as blue. The various mutant promoter constructs were transfected into U937 cells and the expression of luciferase reporter gene was assessed relative to the wild-type pGL3*hres*0.7K construct. Experiments were repeated three times in duplicate.

Having shown the presence of Sp1 cognate motifs and the *in-vitro* binding of Sp1 factor present in the nuclear extracts of U937 cells, we attempted to examine the functional significance of this interaction. Site-directed mutagenesis of the individual transcription factor binding sites was performed and the mutated sequences were cloned upstream of luciferase reporter gene in pGL3 Basic plasmid. A schematic representation of the various mutated constructs is given in Figure 3 (left half). The various mutant constructs were transfected into U937 cells and the expression of luciferase reporter gene was assayed. It could be seen that mutation in AP-1 site did not change the transcriptional activation of luciferase whereas mutations in c-Rel binding site resulted in only a slight decrease in promoter activity compared to full length construct, pGL3*hres*0.7K (Figure 3, right half). A similar decrease in luciferase activity was observed when both AP-1 and c-Rel binding sites were mutated. Interestingly, mutations in the Sp1 binding site of human resistin showed a significant decrease in the expression of luciferase reporter gene. Similarly, mutation of both AP-1 and Sp1 binding sites resulted in a decrease in promoter activity that is comparable to the Sp1 Mut construct (Figure 3). The significant loss of promoter activity in Sp1 mutant construct suggests that binding of Sp1 transcription factor to its cognate motif in TATA-less *hres* gene promoter is an important step in the initiation of transcription and is responsible for maintaining the basal level of resistin gene expression in U937 cells.

In order to address the question whether Sp1 actually induces the transcription of endogenous resistin gene, two sets of experiments were carried out. In the first experiment U937 cells were transfected with plasmid over-expressing Sp1, Sp3 or PPARγ [33] and the expression of human resistin gene was measured under such conditions. It could be clearly seen that upon over-expression with Sp1 or Sp3 (Figure S2, bar 2 and 3), resistin gene transcription was significantly enhanced which was expectedly reduced upon over-expression with PPARγ (Figure S2 bar 4). In parallel experiment, HEK cells were co-transfected with plasmid constructs carrying resistin gene promoter, with or without Sp1 cognate motif, along with Sp1 over-expression plasmid and the expression of luciferase reporter gene was monitored. It could be clearly seen that luciferase expression was significantly increased when Sp1 was also over-expressed (Figure S3A compare bar 2 with bar 1). However, when the Sp1 motif was mutated no such Sp1 mediated enhancement in luciferase gene was seen (Figure S3B, compare bar 2 and 3 with bar 1). These results convincingly demonstrate that Sp1 is able to cause enhanced expression of resistin gene not just under *in vitro* conditions but also *in vivo*.

### Sp1 interacts with PPARγ to determine the constitutive expression of human resistin in U937 cells

**Figure 4:**
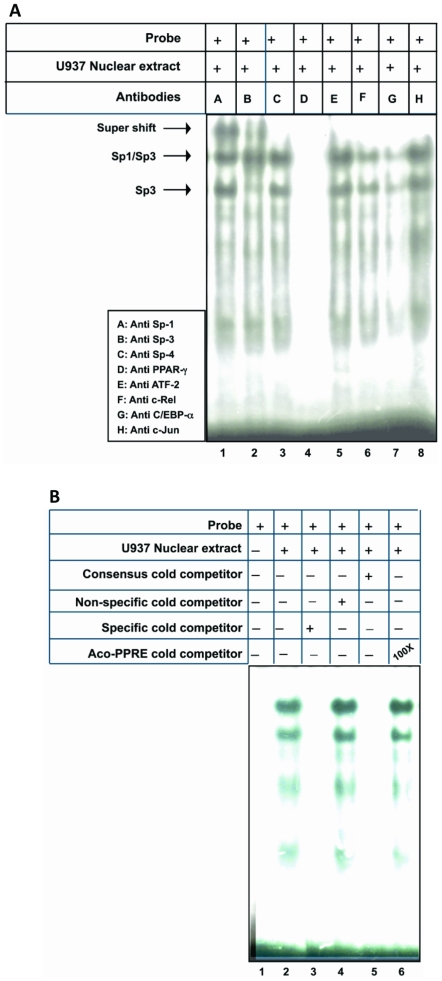
A. PPARγ recruits Sp1 and forms a complex. Super-shift using antibodies as indicated in the corresponding lanes are listed in the inset. Arrows show the binding and the super-shifts. B. PPARγ does not show binding to the Sp1 binding site in human resistin gene promoter. Experiments were repeated three times.

Having determined that Sp1 is the major transcriptional regulator of resistin gene expression in U937 cells, it was important to identify other interacting partners of Sp1, since Sp1 is known to cross-talk with the basal transcriptional machinery at the TATA-less promoter [31], [34] as well as with transcription factors bound to either activator or enhancer sequences [35]–[38]. EMSA was performed using radio labeled *hres* Sp1 oligonucleotide and nuclear extracts prepared from U937 cells. The identity of the factors present in the protein-DNA complex was determined using super-shift with anti-Sp1, anti-Sp3, anti-Sp4, anti- PPARγ, anti-ATF-2, anti-c-Rel, anti-C/EBP-α and anti-c-Jun antibodies (Figure 4A, lanes 1 to 8, respectively). Anti-Sp1 antibodies (lane 1) and anti-Sp3 antibodies (lane 2) super-shifted the protein-DNA complex. No super-shift was observed in the presence of antibodies against Sp4 (lane 3), ATF-2 (lane 5), c-Rel (lane 6), C/EBP-α (lane 7) or c-Jun (lane 8). These observations indicate that Sp1 interacts with Sp3, but not with the other transcription factors, to regulate resistin gene expression. Interestingly, when PPARγ antibody was used (lane 4), the Sp1-Sp3 heterodimer as well as the Sp3 homodimer complex formation was completely abolished. The disappearance of the protein-DNA complex in the presence of anti PPARγ antibodies indicates an interaction of PPARγ with Sp1 and Sp3.

To further document the interaction of PPARγ with Sp1 through Sp1 cognate motif present within *hres* regulatory sequences, EMSA was performed using *hres* Sp1 oligonucleotide. Once again the authenticity of Sp1 complex (Figure 4B, lane 2) is evident from competition with specific homologous (lane 3) and consensus (lane 5) unlabelled oligonucleotide but not when non-specific oligonucleotide (lane 4) was used in competition assays. Interestingly, 100× molar excess of unlabelled Aco-PPRE (PPARγ Response Element from Acyl-CoA oxidase gene promoter) oligonucleotide (lane 6) and also 500× molar excess (data not shown) had no effect on the protein-DNAcomplex. This observation establishes that while PPARγ does not bind to Sp1 recognition sequence within the *hres* gene promoter, complete disappearance of the complex with anti-PPARγ antibodies (Figure 4A, lane 4) may be due to the physical interaction between Sp1 and PPARγ.

### PMA induces resistin gene expression by increasing the recruitment of Sp1 and Sp3 to its cognate motif in human resistin gene promoter

**Figure 5:**
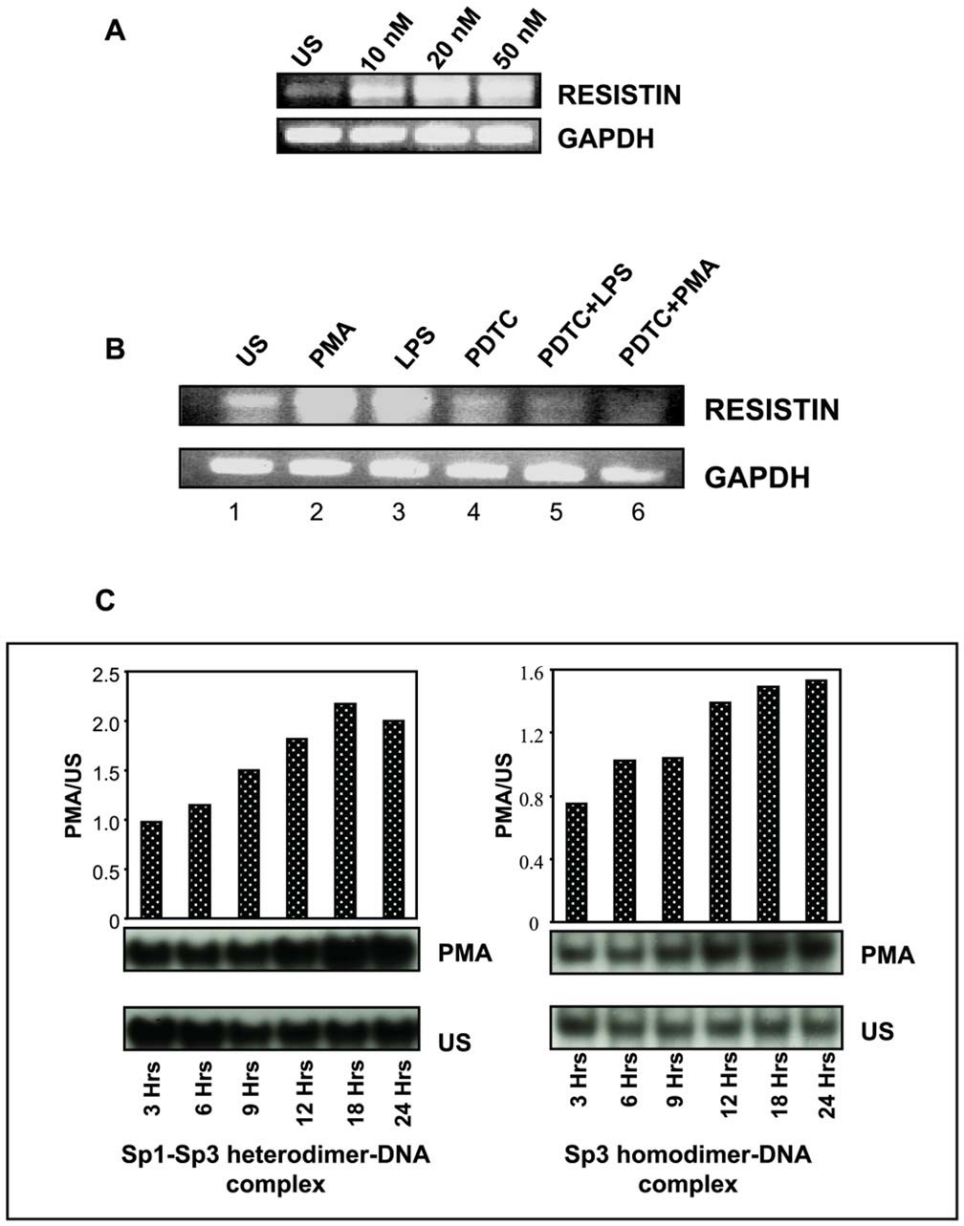
(A,B) PMA and LPS are positive regulators of resistin gene expression and PTDC is a negative regulator as evident from RT-PCR. U937 cells were treated with increasing concentrations of PMA for 3 hours. Quantitative RT-PCR was performed with total RNA isolated from the cells. B. PMA induces *hres* expression while PDTC downregulates it. U937 cells were treated with 100 nM PMA and 5 µg/ml. LPS in the presence or absence of 1 mM PDTC for 3 hours. Quantitative RT-PCR was performed to analyze the effect on resistin gene expression. C. **PMA enhances the recruitment of Sp1 and Sp3 transcription factors to the consensus Sp1 binding site.** Sp1 activity assay using nuclear extract prepared at different time points from PMA stimulated U937 cell. The experiments were repeated at least two times.

Earlier reports have shown that resistin is induced during the differentiation of monocyte to macrophage [10]. Given the fact that PMA is an inducer of monocyte differentiation to macrophages, the effect of PMA on resistin gene expression was determined in U937 cells. LPS has been reported to increase the expression of human resistin gene [11], and was used as a positive control. We therefore first established that PMA is a potent stimulator of resistin gene expression in a concentration dependent manner (Figure 5A). Human monocytic U937 cells were stimulated with PMA and LPS in presence and absence of PDTC, an inhibitor of Sp1 [39]. Quantitative RT-PCR analysis revealed that PMA and LPS could stimulate the expression of resistin (Figure 5B, lanes 2 and 3) while PDTC negatively regulates resistin gene expression (Figure 5B, lane 4). Further, PDTC could inhibit both LPS and PMA induced *hres* gene expression (Figure 5B, lanes 5 and 6, respectively). These results point to Sp1 mediating the PMA and LPS induced human resistin gene expression *in vivo* in U937 cells which is abrogated in the presence of PDTC, an inhibitor of Sp1.

Having observed that PDTC, which is an inhibitor of Sp1, decreases the basal, LPS- and PMA-induced resistin mRNA expression, it was of interest to examine the recruitment of Sp1 to the *hres* gene promoter. Cells were treated with 5 ng/ml PMA and nuclear extract was prepared at various time points as indicated in Figure 5C. EMSA was performed using consensus Sp1 oligonucleotide as the radio labeled probe. It could be seen that with increasing time there was an increased formation of Sp1-Sp3 heterodimer as well as Sp3 homo-dimer complex (Figure 5C). This increase in protein- DNA interaction corresponded to the PMA induced differentiation of U937 monocytes to macrophages. Hence, it can be concluded that Sp1 and Sp3 transcription factors are activated during PMA induced differentiation of monocytes and thus consequently up regulate resistin gene expression during differentiation of U937 monocytes.

### PMA abolishes the interaction of Sp1 with PPAR

**Figure 6:**
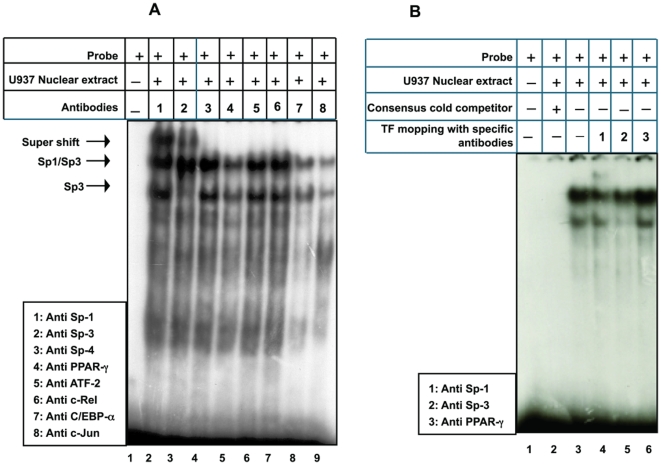
PMA abolishes the interaction of PPARγ with Sp1. A. Super-shift assay with antibodies to Sp1, Sp3, Sp4, PPARγ, ATF-2, c-Rel, C/EBP-α and c-Jun using nuclear extracts prepared from PMA stimulated U937 cells. No super-shifting of the complex was observed in the presence of PPARγ antibody. Note that in lane 5 where PPARγ antibodies were used, the Sp1-Sp3 as well as the Sp3 complex was intact. B. Partial removal of PPARγ from U937 nuclear extract does not affect the recruitment of Sp1-Sp3 complex. EMSA with U937 nuclear extracts depleted of Sp1, Sp3 and PPARγ as indicated. Experiments were done twice.

PMA is known to activate Sp1 dependent transcription [40]. Further, PPARγ has been implicated in the differentiation of human macrophages [20], [41]–[43]. Hence, it was of interest to explore the nature of interaction between Sp1 and PPARγ in *hres* gene promoter upon stimulation with PMA. EMSA was performed using *hres* specific Sp1 oligonucleotide as radio labeled probe and nuclear extracts from PMA stimulated U937 cells (Figure 6A). The identity of the factors present in the protein-DNA complex was determined by super-shift assay using anti-Sp1, anti-Sp3, anti-Sp4, anti-PPARγ, anti-ATF-2, anti- c-Rel, anti-C/EBP-α and anti-c-Jun antibodies (Figure 6A, lanes 2 to 9). Anti-Sp1 and anti-Sp3 antibodies could bring about a super-shift of the DNA-protein complex (Figure 6A, lanes 2, 3) demonstrating that PMA does not affect the Sp1- Sp3 interaction. No super-shift was observed in presence of either Sp4, PPARγ, ATF-2, c-Rel, C/EBP-α or c-Jun antibodies (Figure 6A, lanes 4, 6, 7, 8 and 9, respectively). Interestingly, unlike what was observed in the unstimulated nuclear extracts (Figure 4A, lane 4), where the formation of the protein-DNA complex was completely abolished when PPARγ antibodies were used for super-shift, the Sp1-Sp3 as well as the Sp3 complexes were intact in PMA stimulated U937 nuclear extract (Figure 6A, lane 5). This clearly suggests that the interaction of Sp1 with PPARγ is abolished in the PMA stimulated U937 nuclear extracts.

To further confirm that the integrity of the protein-DNA complex in PMA stimulated U937 nuclear extract is due to lack of physical interaction between PPARγ and Sp1 in PMA stimulated U937 cells, specific antibodies against Sp1, Sp3 and PPARγ were used to selectively deplete these transcription factors from unstimulated U937 nuclear extracts. Nuclear extracts from unstimulated U937 cells were incubated individually with antibodies against Sp1, Sp3 and PPARγ followed by mopping of the immuno-complex with Protein G sepharose beads. These depleted nuclear extracts were used for EMSA (Figure 6B). Decreased binding of Sp1 and Sp3 to the radio labeled probe was observed when Sp1 and Sp3 depleted nuclear extract was used (Figure 6B, compare lane 3 with lanes 4 and 5). Interestingly, it was observed that PPARγ depleted nuclear extract showed a slight increase in binding (Figure 6B, compare lane 3 with lanes 6). This observation suggests that the removal of PPARγ marginally increased the recruitment of Sp1 and Sp3 to the *hres* Sp1 oligonucleotide. Removal of PPARγ from unstimulated U937 nuclear extract mimicked the state of nuclear extract from PMA induced U937 cells where the physical interaction of PPARγ with Sp1 was abolished leading to the enhanced transcriptional activity of resistin gene promoter.

**Figure 7:**
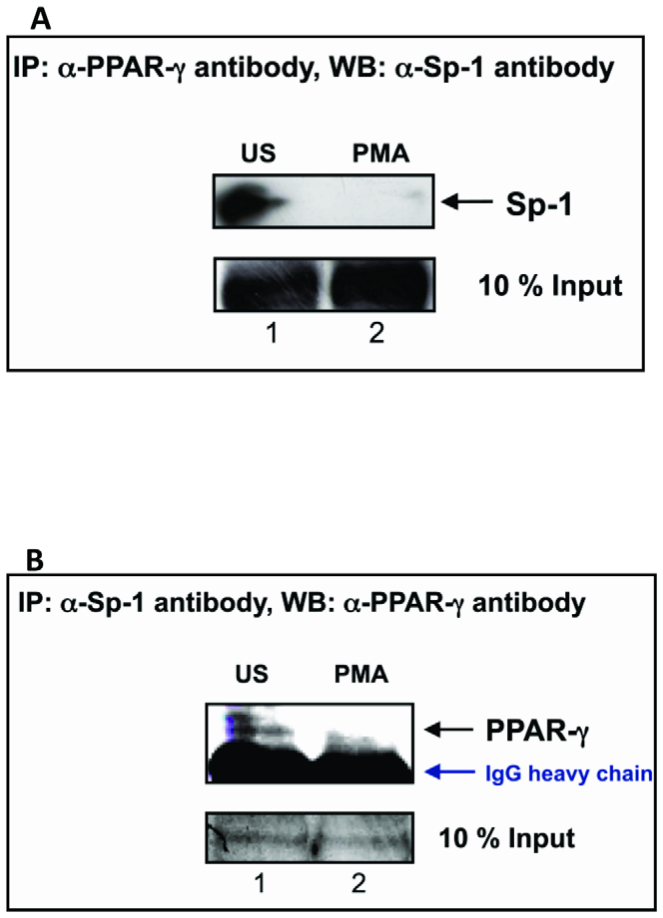
Sp1 and PPARγ transcription factors interact as evident from co-immuno precipitation and western blot analyses. Co-immuno precipitation with Sp1 and PPARγ was performed using cell extracts prepared from PMA stimulated (PMA) or un-stimulated (US) U937 cells. Immuno-precipitates were immuno-blotted with Sp1 and PPARγ antibodies. Bands corresponding to Sp1 and PPARγ are indicated by black arrows. The IgG heavy chain is indicated in blue. This experiment was repeated twice.

Direct interaction between Sp1 and PPARγ was further confirmed by co immuno-precipitation experiments. Whole cell extracts from PMA stimulated or unstimulated U937 cells were immune-precipitated with anti- Sp1 antibodies and immune-blotted with PPARγ antibodies and vice versa (Figure 7). It could be seen that PPARγ co-precipitated with Sp1 in unstimulated extracts (Figure 7A and B, lane 1). Interestingly, neither Sp1 nor PPARγ could be detected when immune-precipitation was carried out using PMA stimulated whole cell extracts (Figure 7A and B, lane 2). These observations indicate that Sp1 interacts directly with PPARγ in unstimulated U937 cells and this interaction is abolished upon stimulation with PMA.

### Functional involvement of Sp1 and PPARγ in human resistin regulation

**Figure 8:**
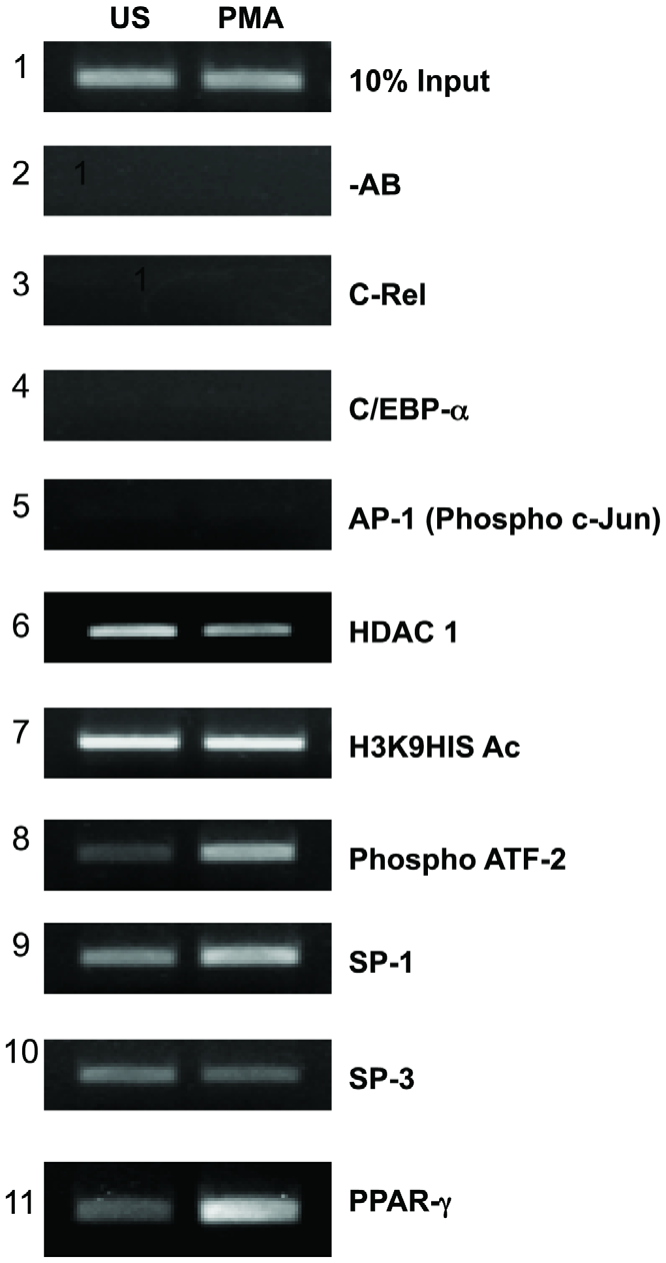
*In vivo* recruitment of transcription factors to resistin gene promoter. ChIP assay was performed with anti c-Rel, anti-C/EBP-α, anti phospho-cJun, anti-HDAC1, anti-acetylated histone H3 (H3K9His Ac), anti phospho-ATF2, anti-Sp1, anti-Sp3 and anti-PPARγ antibodies from PMA treated (PMA) or untreated (US) U937 cells. This experiment was done thrice.

To categorically validate the functional significance of the various transcription factor binding motifs within the *hres* gene promoter, ChIP (Chromatin Immuno-precipitation assay) was carried out. U937 cells were treated with 5 ng/ml PMA for 5 hours and chromatin was immune-precipitated using antibodies specific to different transcription factors. PCR was performed with immune-precipitated DNA to amplify the 535 bp region from −213 to −748 of the *hres* gene promoter (Figure 8). Antibodies to c-Rel (Panel 3), C/EBP- α (Panel 4) and phospho c-Jun (Panel 5) could not immune-precipitate the *hres* gene promoter thereby pointing to the non-essential nature of these transcription factors in *hres* gene regulation.

Immuno-precipitation of the *hres* gene promoter with antibodies to HDAC 1 (Panel 6), acetylated form of histone H3 at lysine 9 (Panel 7), phospho ATF-2 (Panel 8), Sp1 (Panel 9), Sp3 (Panel 10) and PPARγ (Panel 11) points to their role in the transcription of human resistin. It could also be seen that the binding of phospho ATF-2, Sp1 and PPARγ transcription factors to the promoter region was enhanced in PMA treated cells while Sp3 recruitment to the promoter remained unchanged (Figure 8). Further, the recruitment of HDAC 1 to the *hres* gene promoter is decreased in the presence of PMA. Interestingly, no change in the acetylation state of histone H3 was observed in presence of PMA signifying the active state of human resistin gene promoter.

## Discussion

In this paper we describe the nucleotide sequences important for transcription of the human resistin gene which is predominantly expressed in macrophages and has been correlated with inflammation [11]–[15], [44]. The optimal promoter was contained within 80 nucleotide sequence spanning region −238 to −318. *In silico* analysis predicted a putative c-Rel and a Sp1 binding site within the *hres* upstream sequence, along with several other putative binding sites for transcription factors including CEBP-α, AP-1 and ATF-2. Although binding of AP-1 to its cognate motif was observed, site-directed mutagenesis followed by transient transfection did not show significant change in promoter activity. Further, ChIP analysis also did not reveal recruitment of AP-1 to the *hres* gene promoter, confirming the absence of any direct role of AP-1 in the constitutive expression of human resistin. Similarly, site-directed mutagenesis of the c-Rel binding motif did not significantly alter *hres* gene promoter activity. The slight decrease in the promoter activity in c-Rel mut construct possibly could be due to the overlap of the c-Rel binding site with the Sp1 binding site thereby causing reduced recruitment of Sp1. Results from ChIP analysis confirmed that c-Rel may not have any direct role in *hres* gene transcription.

It has been shown that along with C/EBP-α [21], binding of PPARγ to a distant enhancer region was responsible for the increased expression of mouse resistin in adipocytes [23]. The absence of this synergistic action of the C/EBP-α and PPARγ involving the ∼8.8 kb upstream enhancer element in the case of human resistin has been attributed as the reason for differential tissue tropism [23]. Although it was earlier shown, using mouse pre-adipocyte 3T3L1, that C/EBP-α is involved in regulation of human resistin [22] our study did not show any evidence of the same when human monocytic U937 cells were used. The DNA-protein complex seen in EMSA using the putative binding site of C/EBP-α (−221 to −233) as a probe could neither be competed out with consensus C/EBP-α oligonucleotide nor could be super-shifted using anti-C/EBP-α antibodies (Figure S1). Thus C/EBP-α is apparently not relevant for transcription from the *hres* gene promoter despite the presence of a putative C/EBP” binding motif in *hres*. That the nucleotide sequence around this motif nonetheless has a role in *hres* transcription was evident from the fact that mutation in this region led to a slight decrease in the luciferase reporter gene expression (data not shown). It is likely that mutation in this C/EBP binding site (which is in close proximity to the initiator (InR) element and the transcription start site) altered the strength of the InR promoter resulting in decreased promoter activity. The fact that there was no recruitment of C/EBP-α to the human resistin gene promoter *in vivo* using ChIP analysis, provided further support to the argument that C/EBP-α is not important for *hres* gene regulation in monocytes.

TATA-less promoters are known to bind basal transcription factor Sp1 or the related Sp2, 3, and 4 factors through GC-rich sequences which is essential for initiation of transcription [29], [30], [45]. Sp1 and Sp3 compete for binding to GC-boxes and display differential activity, including parallel or opposing transcription effects depending on the promoter [46]–[54] or cell type [29]. That Sp1 and Sp3 bind to *hres* regulatory sequences was further confirmed by site directed mutagenesis of Sp1 binding site and ChIP assay. PMA, an inducer of differentiation of monocyte to macrophage, enhanced the expression of *hres* in U937 cells, as also reported earlier [10]. PDTC, an inhibitor of Sp1, not only suppressed the basal expression of *hres* but also abolished both PMA and LPS induced *hres* expression, suggesting that PMA and LPS mediated induction of resistin is Sp1-dependent. It is noteworthy that PMA induces the recruitment of Sp1 to the target promoter. PMA induced *hres* expression in U937 cells was characterized by an increased recruitment of Sp1 and ATF-2, decreased recruitment of HDAC 1 on resistin gene promoter and no change in the acetylated form of histone H3.

Sp1 facilitates binding of TFIID through TBP Associated Factors (TAFs) to the promoter, which in turn, recruit RNA polymerase II transcription machinery [31], [32], [55]–[58]. Although the activity of Sp1 is generally believed to be constitutive, it may have synergistic effect with many cell type-specific transcription factors such as E2F [35], HNF3 [36], RXR [37], SREBP-1 [59], NF-κB [60] and AP2 [61]. The interaction of PPARγ with Sp1 and Sp3 is important in the regulation of many genes [62]–[65]. TZDs reduce resistin gene expression and this action is believed to be mediated through PPARγ [21]. The role of PPARγ in regulating mouse resistin gene expression through the intronic sequences was earlier shown by us [26]. That PPARγ binds to an enhancer located ∼8.8 kb upstream of the mouse resistin gene transcriptional start site and this confers adipogenic expression of mouse resistin has recently been reported [23]. However, nothing is known about the mechanism of PPARγ mediated regulation of human resistin. In our *in-silico* analysis we could not find PPARγ response elements (PPRE) in the 2.3 Kb upstream or even up to ∼8.8 kb upstream region of *hres* gene [22]. Expectedly, while there was no evidence of direct interaction between PPARγ and *hres* regulatory sequences, surprisingly PPARγ antibodies could abolish the binding of Sp1 and Sp3. Additionally, a physical interaction between PPARγ and Sp1 or Sp3 was evident from co-immunoprecipitation experiments.

Differentiation of U937 cells with PMA disrupted the Sp1-PPARγ interaction *in vitro* although increased *in vivo* recruitment of both Sp1 and PPARγ to the resistin gene promoter was evident from ChIP assay. Hence it is likely that PPARγ interaction with Sp1 negatively regulates *hres* gene expression and abolishment of this interacting during PMA treatment stimulates *hres* expression. It has been reported earlier that thiazolidinediones (TZDs), could not repress the expression of resistin gene in the presence of mithramycin A (Sp1 binding inhibitor) in 3T3-L1 adipocytes [66]. Further it was observed that the level of O-glycosylation of Sp1 was decreased in response to TZDs. Sp1 was shown to be modified by phosphorylation of serine and threonine residues [67]. In addition to phosphorylation, Sp1 also has multiple potential O-glycosylation sites that may be modified by N-acetylglucosamine residues [68]. Recent studies show that Sp1 phosphorylation is not a constitutive modification and is altered in response to extracellular stimuli through a variety of signal transduction pathways. In addition, PPARγ is also able to undergo phosphorylation through various signaling pathways. Taken together, it could be possible that the PMA induced loss of interaction between Sp1 and PPARγ might be due to changes in the dynamic post-translational modifications in either Sp1 or PPARγ. That analyses of such post-translational modifications will lead to a better understanding of the regulation of basal as well as induced expression of human resistin cannot be understated.

In summary, this paper identifies the *cis* acting elements and the role of some of the trans-acting factors in regulating human resistin gene expression. While C/EBP-α is essential for the expression of mouse resistin, it does not appear to be important for human resistin gene expression unlike the expression of mouse resistin gene which is regulated by an enhancer containing PPARγ binding site the discovery of a physical interaction between Sp1 transcription factors and PPARγ, in human macrophages, explains how PPARγ could regulate human resistin gene expression in spite of the absence of its cognate binding site in the resistin gene promoter.

## Supporting Information

Text S1 Supplementary material.
(0.03 MB DOC)

Figure S1 C/EBP-α does not bind to its cognate motif present within the human resistin regulatory sequences. EMSA was performed using nuclear extracts prepared from U937 cells. Radio labeled oligonucleotides containing the binding site for C/EBP-a was incubated with 7.5 µg of nuclear extract and the protein-DNA complex separated on a 7% TGE-acrylamide gel. Lane 1 shows the free probe. Lane 2 is the binding of human resistin C/EBP-α oligonucleotide to C/EBP-α. Lanes 3 and 4 show self and non-self competition respectively. Lanes 5 and 6 show competition with the consensus C/EBP-α oligonucleotide. Lane 7 contains antibodies to C/EBP-α along with the nuclear extract and radio labeled probe. Note that the radio labeled protein-DNA complex formation could not be abolished even in the presence of 200× consensus cold competitor. Note that the DNA-Protein complex could not be super shifted in the presence of anti-C/EBP-α antibodies. Human resistin C/EBP oligonucleotide was used as the radiolabeled probe and human resistin AP-1 oligonucleotide was used for non-self competition.
(0.18 MB TIF)

Figure S2 Endogenous Resistin expression is enhanced both by Sp-1 and Sp-3 over expression but not by PPARγ 2 million U937 cells were transfected with pCMV Sp1 (lane 2), pCMV Sp-3 (lane 3), PPARγ (lane 4) over expression plasmids. Untransfected U937 cells served as control (Lane 1). 48 h after transfection, the cells were harvested and RNA was isolated and semi-quantitative RT-PCR was carried out with 1 µg RNA and resistin primers (forward primer: accggctgcacttgtggctc; reverse primer: cgacctcagggctgcacacg) for 35 cycles at 55°C annealing temperature using RT-PCR kit (Qiagen). GAPDH (forward primer: gcaccaccaactgctta; reverse primer: ccctgttgctgtagccaaat) was used as house keeping control. Histogram was plotted with the ratio of intensities of resistin to GAPDH as determined by ImageJ software.
(0.28 MB TIF)

Figure S3 Sp-1 transcription factor activates transcription from resistin gene promoter.1 million HEK cells were transfected with 1 µg of pGLHres 0.34 k (Bar1) or pGLHres 0.34 k Sp-1mut either alone (Fig B Bar2) or with Sp-1 over expression plasmid (Bar 3) as described. The cells were harvested after 48 h of transfection and processed for luciferase activity.
(0.83 MB TIF)
