# Supplementary material for: Transcription of Human Resistin Gene Involves an Interaction of Sp1 with Peroxisome Proliferator-Activating Receptor Gamma (PPARγ)
Source: PLoS One. 2010 Mar 29;5(3):e9912. doi: 10.1371/journal.pone.0009912 (PMC2848011; doi:10.1371/journal.pone.0009912)
Supplement: Text S1 — Supplementary material. (0.03 MB DOC) [file pone.0009912.s001.doc]

**SUPPLEMENTARY MATERIAL**

**Site-directed mutagenesis of the promoter constructs**

*AP-1 Mut construct*

Mutation in the AP-1 binding site of pGLhres0.7K plasmid was carried out by PCR amplification using 5’ GGGGTACCAGTGCAGTGCTGTGATCATAAAACACTG 3’ as forward primer and

5’CCCAAGCTTACAGGGAGGAGGAGGAGACAGAG 3’ as the reverse primer. The amplified product was digested with *Kpn*I + *Hin*dIII and ligated into *Kpn*I and *Hin*dIII sites of pGL3 Basic plasmid to generate the AP-1 Mut construct.

*c-Rel Mut* and *Sp1 Mut* constructs

Mutations in c-Rel and Sp1 binding sites were obtained using pGLhres0.7K plasmid as the backbone for PCR amplification. The primer pairs used for amplification are 5’

TTGGGGGCCCAGGGACT3’ and 5’CCCAAGCTTACAGGGAGGAGGAGGAGACAGAG3’ for c-Rel Mut and 5’

TTGGGGGCCCAGGGACTTATTAGCCAAGCCAGGAAGCCCCATTCCAAG3’ and

5’CCCAAGCTTACAGGGAGGAGGAGGAGACAGAG 3’ for Sp1 Mut. pGLhres0.7K plasmid was digested with *Kpn*I and *Apa*I and the fragment released was ligated with *Apa*I and *Hin*dIII digested *c-Rel Mut* and *Sp1 Mut* amplicons. These ligated fragments were purified and re-ligated into *Kpn*I and *Hin*dIII digested pGL3Basic plasmid to generate *c-Rel Mut* and *Sp1 Mut* constructs.

*AP-1 + C-rel Mut and AP-1 + Sp1 Mut constructs*

Mutations in c-Rel and Sp1 binding sites were obtained using the strategy described above. *AP-1 Mut* plasmid was digested with *Kpn*I and *Apa*I and the fragment released was ligated with *Apa*I and *Hin*dIII digested *c-Rel Mut* and *Sp1 Mut* amplicons. These ligated fragments were purified and re-ligated into *Kpn*I and *Hin*dIII digested pGL3Basic plasmid to generate *AP-1 + c-Rel Mut* and *AP-1 + Sp1 Mut* constructs. All mutations were confirmed by sequencing.

*Transient transfection*

U937 cells were transfected with 1μg each of the over expression plasmids pCMVSp1, pCMVSp3 and pCMVPPARγ using lipofectamine 2000 (Invitrogen) essentially following the manufacturers protocol with slight modification. pBSK was used as filler plasmid to equalize DNA concentration. Transfected cells were harvested and total RNA was isolated using TRIzol (Invitrogen), RNA estimation was done by Nanodrop (). RT PCR was performed for resistin gene using gene specific primers.

HEK cells were transfected with reporter constructs pGLhres 0.34K or pGLhres 0.34K-Sp1Mut either alone or with pCMVSp1 (Addgene) using lipofectin (Invitrogen) as per the manufacturer’s instruction. Cells were harvested after 48 hours post transfection and lysed in reporter lysis buffer and processed for luciferase assay as per manufacturer’s instruction (Promega). Protein estimation was done by BCA (Pierce) for normalization. Experiment was repeated three times.

**Supplementary Figure 1. C/EBP-α does not bind to its cognate motif present within the human resistin regulatory sequences.** EMSA was performed using nuclear extracts prepared from U937 cells. Radio labeled oligonucleotides containing the binding site for C/EBP-α was incubated with 7.5 μg of nuclear extract and the protein-DNA complex separated on a 7% TGE-acrylamide gel. Lane 1 shows the free probe. Lane 2 is the binding of human resistin C/EBP-α oligonucleotide to C/EBP-α. Lanes 3 and 4 show self and non-self competition respectively. Lanes 5 and 6 show competition with the consensus C/EBP-α oligonucleotide. Lane 7 contains antibodies to C/EBP-α along with the nuclear extract and radio labeled probe. Note that the radio labeled protein-DNA complex formation could not be abolished even in the presence of 200 X consensus cold competitor. Note that the DNA-Protein complex could not be super shifted in the presence of anti-C/EBP-α antibodies. Human resistin C/EBP oligonucleotide was used as the radiolabeled probe and human resistin AP-1 oligonucleotide was used for non-self competition.

**Supplementary Figure 2. Endogenous Resistin expression is enhanced both by Sp-1 and Sp-3 over expression but not by PPARγ**

2 million U937 cells were transfected with pCMV Sp1 (lane 2), pCMV Sp-3 (lane 3), PPARγ (lane 4) over expression plasmids. Untransfected U937 cells served as control (Lane 1). 48h after transfection, the cells were harvested and RNA was isolated and semi-quantitative RT-PCR was carried out with 1μg RNA and resistin primers (forward primer: accggctgcacttgtggctc; reverse primer: cgacctcagggctgcacacg) for 35 cycles at 55^0^C annealing temperature using RT-PCR kit (Qiagen). GAPDH (forward primer: gcaccaccaactgctta; reverse primer: ccctgttgctgtagccaaat) was used as house keeping control. Histogram was plotted with the ratio of intensities of *resistin* to *GAPDH* as determined by ImageJ software.

**Supplementary Figure 3 Sp-1 transcription factor activates transcription from resistin gene promoter.**1 million HEK cells were transfected with 1 μg of pGLHres 0.34k (Bar1) or pGLHres 0.34k Sp-1mut either alone (Fig B Bar2) or with Sp-1 over expression plasmid (Bar 3) as described. The cells were harvested after 48h of transfection and processed for luciferase activity.
