# Supplementary figures and images for: Transcription of Human Resistin Gene Involves an Interaction of Sp1 with Peroxisome Proliferator-Activating Receptor Gamma (PPARγ)
Source: PLoS One. 2010 Mar 29;5(3):e9912. doi: 10.1371/journal.pone.0009912 (PMC2848011; doi:10.1371/journal.pone.0009912)

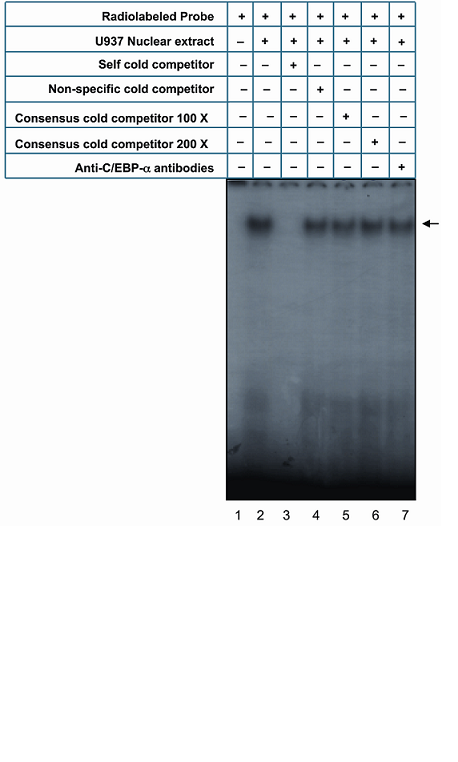

Supplement: Figure S1 — C/EBP-α does not bind to its cognate motif present within the human resistin regulatory sequences. EMSA was performed using nuclear extracts prepared from U937 cells. Radio labeled oligonucleotides containing the binding site for C/EBP-a was incubated with 7.5 µg of nuclear extract and the protein-DNA complex separated on a 7% TGE-acrylamide gel. Lane 1 shows the free probe. Lane 2 is the binding of human resistin C/EBP-α oligonucleotide to C/EBP-α. Lanes 3 and 4 show self and non-self competition respectively. Lanes 5 and 6 show competition with the consensus C/EBP-α oligonucleotide. Lane 7 contains antibodies to C/EBP-α along with the nuclear extract and radio labeled probe. Note that the radio labeled protein-DNA complex formation could not be abolished even in the presence of 200× consensus cold competitor. Note that the DNA-Protein complex could not be super shifted in the presence of anti-C/EBP-α antibodies. Human resistin C/EBP oligonucleotide was used as the radiolabeled probe and human resistin AP-1 oligonucleotide was used for non-self competition. (0.18 MB TIF) [file pone.0009912.s002.tif]

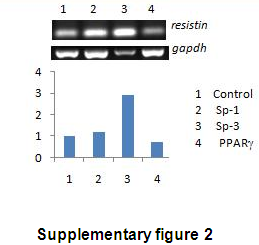

Supplement: Figure S2 — Endogenous Resistin expression is enhanced both by Sp-1 and Sp-3 over expression but not by PPARγ 2 million U937 cells were transfected with pCMV Sp1 (lane 2), pCMV Sp-3 (lane 3), PPARγ (lane 4) over expression plasmids. Untransfected U937 cells served as control (Lane 1). 48 h after transfection, the cells were harvested and RNA was isolated and semi-quantitative RT-PCR was carried out with 1 µg RNA and resistin primers (forward primer: accggctgcacttgtggctc; reverse primer: cgacctcagggctgcacacg) for 35 cycles at 55°C annealing temperature using RT-PCR kit (Qiagen). GAPDH (forward primer: gcaccaccaactgctta; reverse primer: ccctgttgctgtagccaaat) was used as house keeping control. Histogram was plotted with the ratio of intensities of resistin to GAPDH as determined by ImageJ software. (0.28 MB TIF) [file pone.0009912.s003.tif]

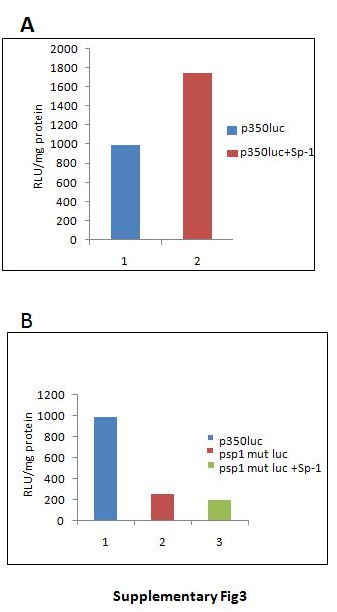

Supplement: Figure S3 — Sp-1 transcription factor activates transcription from resistin gene promoter.1 million HEK cells were transfected with 1 µg of pGLHres 0.34 k (Bar1) or pGLHres 0.34 k Sp-1mut either alone (Fig B Bar2) or with Sp-1 over expression plasmid (Bar 3) as described. The cells were harvested after 48 h of transfection and processed for luciferase activity. (0.83 MB TIF) [file pone.0009912.s004.tif]
